# “Noisy beets”: impact of phenotyping errors on genomic predictions for binary traits in *Beta vulgaris*

**DOI:** 10.1186/s13007-016-0136-4

**Published:** 2016-07-18

**Authors:** Filippo Biscarini, Nelson Nazzicari, Chiara Broccanello, Piergiorgio Stevanato, Simone Marini

**Affiliations:** Department of Bioinformatics and Biostatistics, PTP Science Park, Via Einstein - Loc. Cascina Codazza, 26900 Lodi, Italy; Council for Agricultural Research and Economics (CREA), Research Centre for Fodder Crops and Dairy Productions, Lodi, Italy; DAFNE, Università di Padova, Padua, Italy; Bioinformatics Center, Institute for Chemical Research, Kyoto University, Kyoto, Japan

**Keywords:** Noisy data, Classification, K-nearest neighbours (KNN), Random forest (RF), Support vector machines (SVM), Ridge logistic regression, Sugar beet, Binomial phenotype, Robustness to errors, Genomic predictions

## Abstract

**Background:**

Noise (errors) in scientific data is endemic and may have a detrimental effect on statistical analyses and experimental results. The effects of noisy data have been assessed in genome-wide association studies for case-control experiments in human medicine. Little is known, however, on the impact of noisy data on genomic predictions, a widely used statistical application in plant and animal breeding.

**Results:**

In this study, the sensitivity to noise in the data of five classification methods (K-nearest neighbours—KNN, random forest—RF, ridge logistic regression—LR, and support vector machines with linear or radial basis function kernels) was investigated. A sugar beet population of 123 plants phenotyped for a binary trait and genotyped for 192 SNP (single nucleotide polymorphism) markers was used. Labels (0/1 phenotype) were randomly sampled to generate noise. From the base scenario without errors in the labels, increasing proportions of noisy labels—up to 50 %—were generated and introduced in the data.

**Conclusions:**

Local classification methods—KNN and RF—showed higher tolerance to noisy labels compared to methods that leverage global data properties—LR and the two SVM models. In particular, KNN outperformed all other classifiers with AUC (area under the ROC curve) higher than 0.95 up to 20 % noisy labels. The runner-up method, RF, had an AUC of 0.941 with 20 % noise.

**Electronic supplementary material:**

The online version of this article (doi:10.1186/s13007-016-0136-4) contains supplementary material, which is available to authorized users.

## Background

Errors in the data collected for scientific experiments or—especially—for routine industrial applications are referred to as noise in the data, and may arise for several reasons (e.g. instrument errors, human errors, environmental noise, inherent randomness in the physical process, corruption of data etc. [1]). Noisy data are a long known problem in statistics (e.g. [2]). In spite of efforts to clean the data and produce good quality datasets [3], a certain amount of noise is bound to persist in the data: this needs to be dealt with, and the impact on results assessed (e.g. [4–6]). In binary classification problems, noisy data typically take the form of mislabeled observations or flipped labels [6]. For instance, the carrier status of recessive mutations (e.g. [7]) may inadvertently be inverted in some individuals; the same could happen in the case of resistance/susceptibility to diseases (e.g. rhizomania in sugar beet [8], where a proportion of resistant plants could be mislabeled as susceptible, and viceversa). These are examples of possible phenotypic errors in binomial traits.

In the field of genomics, the effect of mislabeled observations on the statistical power of genome-wide association studies (GWAS) has been recognized in case-control studies in human medicine [9, 10]. Buyske et al. found that a 39-fold larger sample size is required to maintain the same power of analysis in case-control studies with 5 % misclassifications. In animal genetics, a known issue are pedigree errors and their effect on the accuracy of estimated breeding values [11]: for instance, in a pig population with 20 % errors in the pedigree, the average genetic gain showed a reduction in the range 3.2–12.4 % for a number of traits. Noisy data are bound to have a detrimental effect also on whole-genome predictions, which are increasingly used for a variety of phenotypes in plant and animal breeding [12]. Additionally, the current trend in precision agriculture is bringing about novel high-throughput phenotyping systems to measure a vast amount of data in an automatic and continuous way [13], which may well harbor a certain proportion of errors. Automatically generated non-curated datasets are prone to contain errors (e.g. [14, 15]). There are currently no studies that address the issue of noisy data in genomic predictions, neither in humans, nor in plants and animals.

In this paper, the impact of random noise on the accuracy of genomic predictions for binary traits is investigated. Starting from a population of sugar beet with known binomial phenotypes, increasing proportions of noisy labels were randomly generated, and the performance of different classification methods was measured.

## Methods

### Plant phenotypes and genotypes

In total, 123 sugar beet (*B. vulgaris*) plants were available, 99 with high- and 24 with low-root vigor. Plants were originated from 18 selected sugar beet lines (15 with high- and 3 with low-root vigor). Root vigor is linked to nutrient uptake and plant productivity, [16] and, in selected sugar beet populations, has been usually treated as a binary trait [17, 18]. Classification of plants into high- or low-root vigor was based upon phenotypic measurement of root elongation on 11-day-old seedlings: root elongation was on average 12.9 and 2.6 mm/day in high and low root vigor plants, respectively. The clearly bimodal distribution can be seen in Biscarini et al. 2015 [18].

All plants were genotyped for 192 SNP markers with the high-throughput marker array QuantStudio 12K Flex system coupled with Taqman OpenArray technology. The average per-sample and per-marker call-rate were 0.984 and 0.969. Only one SNP had a per-marker call-rate ${\le }85\,\%$ and was removed. There were in total 738 missing genotypes (3.14 %). After imputation ([19]) data were edited for minor allele frequency (MAF): 16 SNPs with MAF ${\le }2.5\,\%$ were discarded. After editing, 175 SNPs evenly distributed across the nine chromosomes of the sugar beet genome were left for the analysis.

Further description of phenotypes and genotypes can be found in [17, 18, 20–22].

The study was conducted in accordance with the existing national and international guidelines and legislation.

### Classification models

Based on SNP genotypes, the genomic classification of individual sugar beet plants into the two classes (high- and low-root vigor) was carried out using the following five models:

*K-nearest neighbors (KNN) classifier* The predicted class for plant $x_0$ was obtained by majority vote among the *K* closest neighbours. The neighbourhood was determined via Euclidean distances based on SNP genotypes ($D_E=d(x_0,x_i)=\sqrt{\sum _{j=1}^m (x_{0j}-x_{ij})^2}$, for each neighbour *i*, over *m* SNP dimensions). The vote of neighbors could be differentially weighted (or not) by the inverse ($1-D_E$) or the reciprocal $\left( {1}/{D_E} \right)$ of the distance from the unlabelled observation $x_0$. Whether and how to weight neighbouring observations was determined through cross-validation.

*Random forest *(*RF*) *classifier* A large number of classification trees was built on *B* bootstrapped samples of sugar beet plants. Classification trees were decorrelated by using, at each node, a random subset *m* of the 175 SNP. The final classifier was obtained by majority vote over the *B* classification trees:

1 $$\hat{f}_{avg}(x_i)=\frac{1}{B}\sum _{b=1}^B I(\hat{f}_b(x_i)=[0/1])$$

where $x_i$ is the vector of SNP genotypes for plant *i*, and $\hat{f}_b(x_i)$ is the prediction (high-/low-root vigor) from the classification tree built on the $b_{th}$ bootstrapped data sample. More details on random forest can be found in [23].

*Ridge logistic regression (LR) classifier* The probability of having high-root vigor ($P(Y=1|X)=p(x)$) was modeled as a linear combination of the SNP genotypes in a logistic regression model:

2 $$logit(p(x_i))=\mu +\sum _{j=1}^m z_{ij}{\textit{SNP}}_j$$

where $p(x_i)$ is the $P(Y=1|X)$ for individual *i* with vector of SNP genotypes $x_i$; ${\textit{SNP}}_j$ is the effect of the $j_{th}$ marker; $z_{ij}$ is the genotype of individual *i* at locus *j* (0, 1 or 2 for AA, AB and BB genotypes). Since the number of markers in the model (175 SNPs) exceeds the number of observations (123 plants), an $\ell 2$-norm penalization ($-\frac{1}{2} \uplambda \sum _{j=1}^m SNP_j^2$) was applied to the likelihood function to be maximised [24].

*Support vector machine with linear kernel (SVM-Lin)* SVM-Lin maps the vector of SNP genotypes ${\mathbf {x}} \in {\mathbb {R}}$ into a higher dimensional feature space $\phi ({\mathbf {x}}) \in {\mathbb {H}}$ and constructs a separating hyperplane-linear in ${\mathbb {R}}$- to classify observations based on the width of the margin *M* and the sign of the classifier:

3 $$f(x)=\beta _0+\sum _{i=1}^n\alpha _iK(x,x_i)$$

The mapping ${\mathbb {R}} \mapsto {\mathbb {H}}$ is performed by a linear kernel function $K(x_i,x_{i'}) = \langle x_i,x_{i'} \rangle$ which defines an inner product of pairs of SNP genotype vectors in the space ${\mathbb {H}}$. The intercept $\beta _0$ and the coefficients $\alpha _i$ are obtained by maximizing the margin *M*, whose width is controlled by the hyperparameter *C*, optimized through cross-validation.

*Support vector machine with radial basis function kernel (SVM-Rbf)* As in SVM-Lin, observations are classified by the sign of Eq. 3 and the width of margin *M*; only, in SVM-Rbf the kernel function *K* is the radial basis function: $K(x_i,x_{i'})=\exp \left( -\gamma \sum _{j=1}^p(x_{ij}-x_{i'j})^2\right)$. The width of the margin *M* is again controlled by the hyperparameter *C*, while the positive constant $\gamma$ controls the degree of non-linearity of the decision boundary.

For a full description of SVM with either linear or radial basis function kernel, see [25].

### Tuning the hyperparameters, generating noisy labels and measuring classification accuracy

The hyperparameters in the models were optimised through cross-validation among a range of values: for KNN, the number of neighbors $K \in \left\{ 1, 3, 5, 7 \right\}$ and their weight $\in \left\{ 1, 1 - D_E, {1}/{D_E} \right\}$; for LR, the value of the penalty $\uplambda$; in RF the number of *B* “bootstrapped trees” $\in \left\{ 1, 5, 10, 50, 100 \right\}$ and the subset of *m* SNP markers per node $\in \left\{ j, 2, 4 \right\}$, where *j* is $int(log_2(\#\_of\_SNPs) + 1 ))$; in SVM, the cost parameter $C \in \left\{ 2^2 \cdots 2^9 \right\}$ for both SVM-Lin and SVM-Rbf; for SVM-Rbf, additionally, the positive constant $\gamma \in \left\{ 10^{-3} \cdots 10^{+1} \right\}$.

To test the impact of phenotyping errors on genomic predictions, an increasing fraction of the observations in the training set was randomly mislabelled: from 0 % (no mislabels) up to 50 % (theoretical maximum noise), through 12 intermediate steps (1, 2.5, 5, 7.5, 10, 12.5, 15, 17.5, 20, 25, 30, 40 %). At every step, the corresponding fraction of observations was randomly sampled from the original data and the labels were flipped ($0 \rightarrow 1$; $1 \rightarrow 0$). For each proportion of mislabelled observations, the five classification models were tested with a 5-fold cross-validation scheme. 123 sugar beet plants were randomly split into 5 subsets of approximately the same size. In turn, the observations in one subset were set to missing and predicted using the model trained with the remaining four subsets, until all subsets were used once as validation set. A further nested 5-fold cross-validation run was applied for hyperparameter optimization. Labels predicted in the validation set were compared to the original (true) labels to measure the accuracy of classification. Each experiment (proportion of mislabelled observations per classification model) was repeated 100 times ($\times$5-fold cross-validation = 500 replicates). Results were averaged to explore the variability of prediction and ensure numeric stability.

High root vigor (the majority class) was by convention considered *positive* and low root vigor (the minority class) *negative*. The accuracy of genomic predictions was measured as: (1) Total error rate (TER: ratio between the number of classification errors and the total number of predictions), (2) False positive rate (FPR: ratio between wrongly predicted positives and the total predicted positives), and (3) False negative rate (FNR: ratio between false negative predictions over all negative predictions). Additionally, the area under the receiver operating characteristic (ROC) curve (AUC) was also recorded to monitor FPR and FNR over all possible classification thresholds in [0,1] [26].

### Software

All models were implemented using the *Weka* machine learning suite [27]. The open source statistical environment *R* [28] was used generate random noisy labels, to parse results and produce figures and tables.

## Results

 Error rates (TER, FPR, FNR) for the five classification models over all mislabeling proportions are reported in Table 1. In general, very low error rates were observed with no phenotyping errors in the data (base scenario). No errors overall and in both classes with KNN, LR and SVM-Lin, errors below 0.1 % with SVM-Rbf and around 1 % with RF.

**Fig. 1:**
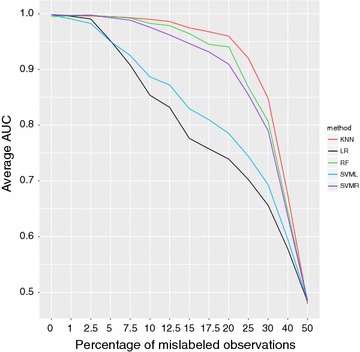
Average area under the ROC curve (AUC) for the five tested classification models as a function of increasing proportions of mislabeled observations. Averages are for validation values from 5-fold cross-validation repeated 100 times. KNN *red line*, RF *green line*, SVM-Lin *blue line*, LR *black line*, SVM-Rbf *violet line*

The average AUC as a function of the proportion of mislabeled observations is a good indicator of the relative performance of the five classification models, and their robustness to noise in the data (Fig. 1). The performance of LR and SVM-Lin decreased approximately linearly with increasing proportions of mislabeled observations. KNN, RF and SVM-Rbf appeared to be more robust to noise in the data: AUC was ${\gtrapprox }0.95$ for KNN and RF, and larger than 0.90 for SVM-Rbf, up to 20 % mislabelled observations: only after 20 % phenotype errors their performance started deteriorating rapidly. With mislabeled observations approaching 50 %, AUC from all classification models quickly converged to 0.50 (absence of any predictive value).

**Fig. 2:**
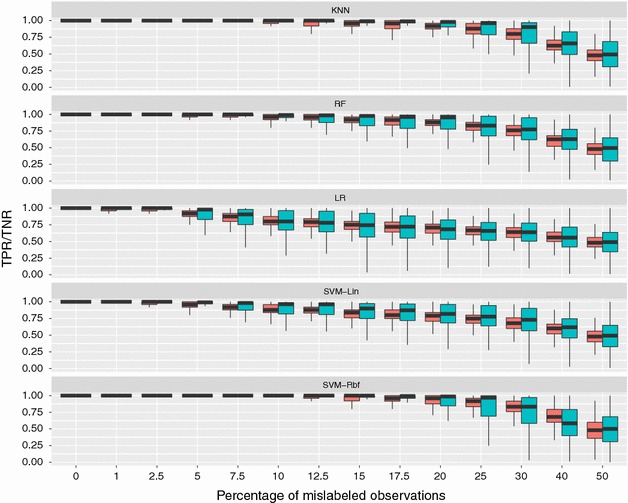
Distribution of TPR (*red*) and TNR (*blue*) in the validation set. TPR and TNR as a function of mislabeled observations, from a 5-fold cross validation repeated 100 times. Results are presented per method

With increasing noise in the data, not only did the average performance decrease, but also the genomic predictions were much more variable. Figure 2 shows the boxplots of the 500 (5-fold cross-validation, repeated 100 times) true positive (*TPR* = 1 − *FNR*) and true negative (*TNR* = 1 − *FPR*) rates per method and proportion of noisy labels. With no or little phenotyping errors classifications were consistently very accurate. With KNN and SVM-Rbf there were virtually no misclassifications up to 7.5 and 10 % mislabeled observations, respectively. With larger fractions of noisy labels, classifications became more unstable and the variability of genomic predictions started spanning the entire range between 0 and 100 % correct classifications.

The low-to-high root vigor ratio was 0.195 in the original data. Mislabeled observations were then generated randomly, and this had an effect on the class ratio, which went up to 0.520 with 50 % noise. When increasing proportions of noise were introduced, data got progressively more balanced. The frequency of the minority class for each proportion of noisy labels is reported in Table 1.

**Table 1:** Total classification error (TER), false negative (FNR) and false positive (FPR) rates, and area under the ROC curve (AUC) for increasing proportions of mislabeled observations with the five tested classification models

misLabels (%)	minFreq	errType	KNN	LR	RF	SVM-Lin	SVM-Rbf
0	0.1950	TER	0.0000	0.0000	0.0085	0.0000	0.0001
		FNR	0.0000	0.0000	0.0067	0.0000	0.0001
		FPR	0.0020	0.0020	0.0054	0.0020	0.0036
		AUC	*1.0000*	0.9980	0.9946	0.9980	0.9961
1	0.1870	TER	0.0039	0.0153	0.0092	0.0077	0.0008
		FNR	0.0042	0.0153	0.0076	0.0078	0.0007
		FPR	0.0038	0.0046	0.0036	0.0095	0.0044
		AUC	0.9961	0.9954	*0.9964*	0.9905	0.9955
2.5	0.1870	TER	0.0045	0.0291	0.0102	0.0145	0.0004
		FNR	0.0049	0.0283	0.0094	0.0139	0.0004
		FPR	0.0041	0.0094	0.0032	0.0174	0.0023
		AUC	0.9959	0.9905	0.9968	0.9825	*0.9977*
5	0.2114	TER	0.0088	0.0897	0.0236	0.0471	0.0047
		FNR	0.0096	0.0864	0.0213	0.0466	0.0043
		FPR	0.0052	0.0484	0.0052	0.0496	0.0073
		AUC	0.9948	0.9516	*0.9951*	0.9503	0.9918
7.5	0.2520	TER	0.0160	0.1431	0.0342	0.0708	0.0087
		FNR	0.0159	0.1386	0.0307	0.0688	0.0077
		FPR	0.0071	0.0920	0.0077	0.0748	0.0116
		AUC	*0.9928*	0.9080	0.9921	0.9251	0.9882
10	0.2439	TER	0.0292	0.2011	0.0553	0.1111	0.0205
		FNR	0.0294	0.1963	0.0521	0.1105	0.0188
		FPR	0.0100	0.1462	0.0173	0.1134	0.0242
		AUC	*0.9898*	0.8538	0.9827	0.8866	0.9754
12.5	0.2846	TER	0.0396	0.2286	0.0679	0.1275	0.0328
		FNR	0.0393	0.2247	0.0625	0.1297	0.0285
		FPR	0.0139	0.1680	0.0214	0.1277	0.0381
		AUC	*0.9861*	0.8320	0.9786	0.8723	0.9614
15	0.2927	TER	0.0536	0.2714	0.0924	0.1687	0.0484
		FNR	0.0533	0.2637	0.0867	0.1687	0.0439
		FPR	0.0254	0.2237	0.0358	0.1705	0.0535
		AUC	*0.9746*	0.7763	0.9642	0.8292	0.9460
17.5	0.2764	TER	0.0691	0.2903	0.1098	0.1887	0.0635
		FNR	0.0692	0.2867	0.1017	0.1889	0.0595
		FPR	0.0323	0.2425	0.0549	0.1903	0.0686
		AUC	*0.9677*	0.7575	0.9451	0.8097	0.9091
20	0.2846	TER	0.0924	0.3095	0.1258	0.2166	0.0835
		FNR	0.0948	0.3068	0.1207	0.2212	0.0767
		FPR	0.0402	0.2608	0.0594	0.2149	0.0906
		AUC	*0.9598*	0.7391	0.9406	0.7851	0.9081
25	0.3984	TER	0.1334	0.3415	0.1947	0.2550	0.1377
		FNR	0.1325	0.3344	0.1829	0.2582	0.1141
		FPR	0.0800	0.2976	0.1320	0.2559	0.1454
		AUC	*0.9198*	0.7024	0.8680	0.7441	0.8532
30	0.3659	TER	0.2073	0.3693	0.2522	0.3079	0.1989
		FNR	0.2079	0.3700	0.2477	0.3156	0.1745
		FPR	0.1518	0.3439	0.1930	0.3067	0.2099
		AUC	*0.8481*	0.6561	0.8069	0.6933	0.7901
40	0.4309	TER	0.3681	0.4382	0.3884	0.4044	0.3551
		FNR	0.3723	0.4376	0.3916	0.4087	0.3088
		FPR	0.3254	0.4223	0.3546	0.4051	0.3639
		AUC	*0.6745*	0.5777	0.6453	0.5949	0.6351
50	0.5203	TER	0.5111	0.5134	0.5194	0.5130	0.5116
		FNR	0.5214	0.5120	0.5199	0.5161	0.5238
		FPR	0.5208	0.5165	0.5170	0.5147	0.5137
		AUC	0.4792	0.4834	0.4830	0.4853	*0.4862*

Reported values of classification performance are average validation results from a 5-fold cross-validation scheme repeated 100 times (per model, per mislabel proportion). MinFreq is the frequency of the minority class (low-root vigor). In italic the best performing method (in terms of AUC) for each percentage of noisy lables

*KNN* K-nearest neighbours, *LR* ridge logistic regression, *RF* random forest, *SVM-Lin* SVM with linear kernel, *SVM-Rbf* SVM with radial basis function

## Discussion

Classifying sugar beet plants into high- and low-root vigor using SNP genotypes was already shown to be very accurate [17, 18]. This provides an excellent starting point, and ensures that observed classification errors are due to noise in the data and the chosen classification model, and not to intrinsic characteristic of the data that could privilege some method over the others.

In general, when noise increases, the rate of misclassifications also increases, together with the variability of genomic predictions, and the two classes gets progressively more balanced (which consequently casues TPR and TNR to get more similar). However, while the classification accuracy of LR and SVM-Lin decreased linearly with the rate of phenotyping errors, KNN, RF and SVM-Rbf were more robust to noise and showed a similar pattern in their AUC curve.

KNN and RF are semi-parametric statistical methods which are inherently “local” in their behaviour, and therefore tend to be robust to outliers in the data. Neighbourhoods (in KNN) and branches (in RF) use subsets of the data and rely on the prevalent labels in the subset to classify observations. It is unlikely that all—or most—mislabelled observations happen to be in one neighbourhood or branch. Therefore KNN and RF would give good performance up to the point when the subset is dominated by misalbeled observation. When the fraction of mislabeled observations is 20 % or higher, the amount of noise is such that probabilities revert, and it gets unlikely to have local subsets without—or with few—mislabelled observations, and also local methods begin to fail [29]. In SVM-Rbf, training observations which are far—in terms of Euclidean distance—from a given test observations $x*$ play essentially no role in predicting the class label of $x*$ ($K(x_i,x_{i'})=\exp \left( -\gamma \sum _{j=1}^p(x_{ij}-x_{i'j})^2\right)$ will in fact be very small [30]). This implies that the SVM-Rbf has a very local behavior, in the sense that only nearby training observations have an effect on the class label of a test observation, similarly to what happens with KNN and RF. This helps explain the similar performance of these three classification methods with increasing noise in the data.

On the other hand, LR and SVM-Lin work very well in the base scenario, when there are no mislabels. This is because in this classification problem the decision boundary is linear, and the two classes are linearly separable (see also phenotypic distribution in the Supplementary Figure SF1 in [18]). With noisy labels, though, LR and SVM-Lin tend to degrade faster than local methods because they build on general properties of the data.

Local classification methods proved to be robust to noise up to 20 % mislabeled observations in the dataset. At this proportion of errors, the average hyperparameters had the following values: for KNN, $\overline{K}=4.49$ (and no weight was used in most of the cases—40 %); for RF, $\overline{B}=37.7$; for SVM-Rbf, $\overline{\gamma }=0.0921$. These hyperparameters control the bias/variance trade-off and their optimization is much dependent on the specific training datasets (e.g. size of the data, number of parameters relative to observations). Therefore, the values of the hyperparameters estimated here are not directly applicable to other datasets, but can provide a guide for the space to be explored in similar problems.

**Fig. 3:**
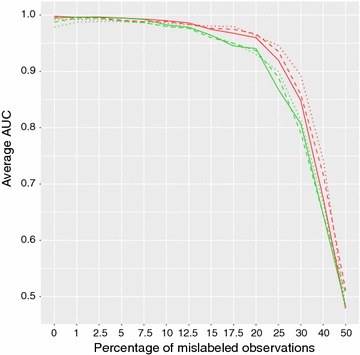
Average area under the ROC curve (AUC) using all 175 SNPs and subsets with the 50 and 30 most informative SNPs. Averages are for validation values from 5-fold cross-validation repeated 100 times. KNN *red lines*, RF *green lines*; All 175 SNP *solid lines*, 50 most informative SNP *dashed lines*, 30 most informative SNP *dotted lines*

Biscarini et al. [18] previously showed that it was possible to reduce the set of markers down to as few as 30 SNP, without losing accuracy of classification. The parsimonious classifier thus developed was here tested with noisy labels. Based on the proportion of variance explained, two subsets with the 50 (SNP50) and 30 (SNP30) most informative SNP loci were extracted and used to classify high- and low-root vigor sugar beets. The two best performing methods were applied: KNN and RF. Figure 3 shows the AUC for increasing proportions of noise in the data when using all 175 SNP or subsets with, respectively, 50 and 30 SNP. The accuracy of classification is practically unaffected by the number of SNP included in the model. The variability of predictions was also little affected: with fewer SNP predictions were only slightly less reliable (e.g. KNN for 5 and 7.5 % noise, see Additional file 1: Figure S1) . These results indicate that informative SNPs appear to be more relevant than sheer SNP density for the accuracy of genomic predictions (e.g. [31]).

Robustness to noise is an aspect of genomic predictions which is currently overlooked, but may be desirable. To extract useful information from data, a classifier that is robust to noisy labels is needed to produce meaningful results even in the presence of noise. There may be interest in methods robust to noise. Manual phenotyping is known to may be prone to errors (e.g. in human medicine [32, 33]). Novel high-throughput phenotyping platforms [34–36], by which very large amounts of data are automatically generated, may alleviate the problem, at least partially. However, automatically generated data are not double-checked for errors, and are therefore susceptible to contain a residual amount of phenotyping errors. This highlights on one hand the importance of accurate phenotyping for genomic predictions [37, 38], on the other the need for prediction methods able to deal with noisy data.

Genomic classification for binary traits is highly relevant in plant breeding (e.g. resistance/susceptibility to diseases [39], which is often controlled by multiple loci e.g. [40]). In sugar beet, besides root vigor, other binomial characteristics of plants are important: for instance bolting tendency (i.e. premature flowering, negatively related to sugar yield [41]), for which a polygenic nature is increasingly evident [42], and genome-enabled predictions promise therefore to be a valuable technique for breeding.

## Conclusions

Noise (errors) is pervasive in scientific data, potentially also in the field of genomics applied to plant breeding. A specific type of errors are misalbeled observations (wrongly assigned labels, flipped labels), which are relevant in the analysis of binary traits. The impact of noisy labels on the accuracy of genome-enabled predictions had not been investigated so far; this paper presented a first attempt at understanding what happens when binary phenotypes are incorrect, and how different classification methods respond to increasing proportions of noisy labels in the data. The results of this study indicate that local classification methods seem to be better suited to cope with noisy labels, with KNN outperforming all other classifiers. Overall, genomic predictions for binomial traits seem to be robust to small percentages of phenotyping errors, and the high variability between methods points at the possibility of selecting the best classifier for each problem, depending on the amount of noise and the nature of the decision boundary.

## Availability of supporting data

SNP genotypes and high/low-root vigor status of the 123 sugar beet samples used in this study are currently not hosted in any open access repository, but are available upon request to the authors.

## Additional file

10.1186/s13007-016-0136-4 TPR/TNR variability with all SNP and with subsets of 30 or 50 SNP Distribution of TPR (red) and TNR (blue) in the validation set using KNN and RF with all 175 SNP and with subsets of 50 and 30 SNP. TPR and TNR as a function of mislabeled observations, from a 5-fold cross validation repeated 100 times. Results are presented per method.

## Abbreviations

SNP: single nucleotide polymorphism
KNN: K-nearest neighbors
RF: random forest
SVM: support vector machines
SVM-Lin: SVM with linear kernel
SVM-Rbf: SVM with radial basis function kernel
AUC: area under the curve
TPR: true positive rate
TNR: true negative rate
