# Supplementary figures and images for: “Noisy beets”: impact of phenotyping errors on genomic predictions for binary traits in Beta vulgaris
Source: Plant Methods. 2016 Jul 18;12:36. doi: 10.1186/s13007-016-0136-4 (PMC4949885; doi:10.1186/s13007-016-0136-4)

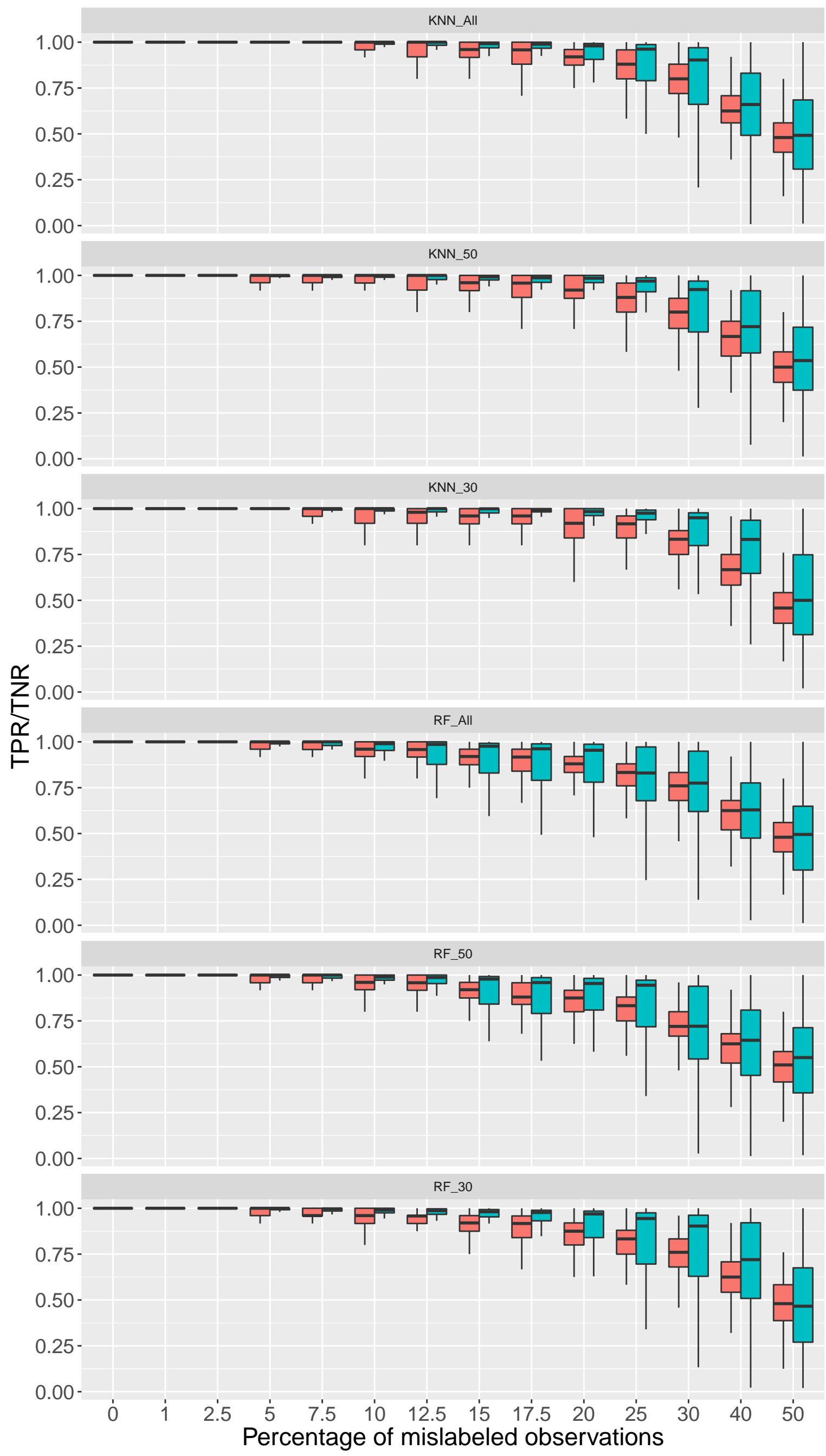

Supplement: Supplementary file 1 — 10.1186/s13007-016-0136-4 TPR/TNR variability with all SNP and with subsets of 30 or 50 SNP Distribution of TPR (red) and TNR (blue) in the validation set using KNN and RF with all 175 SNP and with subsets of 50 and 30 SNP. TPR and TNR as a function of mislabeled observations, from a 5-fold cross validation repeated 100 times. Results are presented per method. [file 13007_2016_136_MOESM1_ESM.pdf]
